# Social networks, social determinants, and mortality: Western New York Exposures and Breast Cancer study

**DOI:** 10.1093/jncics/pkae057

**Published:** 2024-07-17

**Authors:** Shipra Gandhi, Jing Nie, Maurizio Trevisan, Kristopher Attwood, Jo L Freudenheim

**Affiliations:** Roswell Park Comprehensive Cancer Center, Medical Oncology, Buffalo, NY, USA; State University of New York at Buffalo, Epidemiology and Environmental Health, Buffalo, NY, USA; State University of New York at Buffalo, Epidemiology and Environmental Health, Buffalo, NY, USA; College of Health Sciences VinUniversity, Hanoi, Vietnam; Università Campus Biomedico, Rome, Italy; Roswell Park Comprehensive Cancer Center, Medical Oncology, Buffalo, NY, USA; State University of New York at Buffalo, Epidemiology and Environmental Health, Buffalo, NY, USA

## Abstract

**Background:**

There are few studies of social support and other social determinants of health after breast cancer diagnosis and their associations with mortality; results have been inconclusive. Further, it is not known if observed associations are specific to women with breast cancer diagnosis or if associations would be similar among healthy women.

**Methods:**

Women with incident, pathologically confirmed invasive breast cancer, stage I-IV (n = 1012), and healthy frequency age-matched participants (n = 2036) answered a social support questionnaire in prospective follow-up of a population-based case-control study, the Western New York Exposures and Breast Cancer Study. At interview, all participants were aged 35-79 years and resident of 2 counties in Western New York State. Mortality status was ascertained from the National Death Index. Participants were queried regarding the number of their close friends, frequency of seeing them, household size, household income, and marital status. Hazard ratios (HRs) and 95% confidence intervals (CIs) for breast cancer–specific mortality (breast cancer women only) and all-cause mortality were estimated.

**Results:**

Lower household income was associated with higher all-cause mortality among women diagnosed with breast cancer (HR = 2.48, 95% CI = 1.24 to 4.97) and similarly among the healthy women (HR = 2.63, 95% CI = 1.25 to 5.53). Number and frequency of seeing friends, marital status, and household size were not associated with mortality, either among breast cancer patients or among healthy women.

**Conclusion:**

Among those diagnosed with breast cancer and healthy women, lower income was associated with more than twice the mortality. Marital status, household size, and number or frequency of meeting friends were not associated with survival.

Social support, comprising family, friends, and community, is one of the social determinants of health (SDOH), shown to play a role in overall mortality (1,2). In a meta-analysis of 148 studies, there was 50% increased survival for participants with stronger social relationships (1). Social isolation is considered a health problem like other risk factors, including high blood pressure, obesity, and smoking (3). In a comprehensive advisory, the US surgeon general declared loneliness and isolation as health epidemics within the United States (4). Other SDOH, which may also impact availability of social support, include marital status (presence of a spouse or partner) and household size (number of household members). In addition, poor social networks and low social support may be a particular issue for socioeconomically disadvantaged people (5).

Breast cancer is the second leading cause of cancer deaths in women, with an estimated 42 250 deaths in 2024 (6). Breast cancer patients experience psychological and social issues, including psychosocial stress, depression, anxiety, and fear of recurrence, all of which can impact them and their families (7,8). Social and emotional support have been shown to reduce stress and hypothalamic-pituitary-adrenal axis reactivity and decrease cortisol levels (9), which may improve immunosurveillance against cancer recurrence (10).

SDOH, such as poverty, lack of education, neighborhood disadvantage, residential segregation by race, and racial discrimination, play an important role in survival in breast cancer patients (11). Findings regarding associations of social support with breast cancer–specific mortality have been inconsistent (12-15) with considerable limitations, including not accounting for potential confounding by comorbidities and other SDOH. We examined the impact of social support and other related SDOH, marital status, household size, and income on survival, not only among breast cancer patients but also among population-based healthy women. Although prior studies have examined these associations among breast cancer patients and healthy women, no single study has comprehensively examined these associations among both breast cancer patients and healthy women while adjusting for confounding variables in the same analyses.

## Methods

### Study design

**Figure 1.**
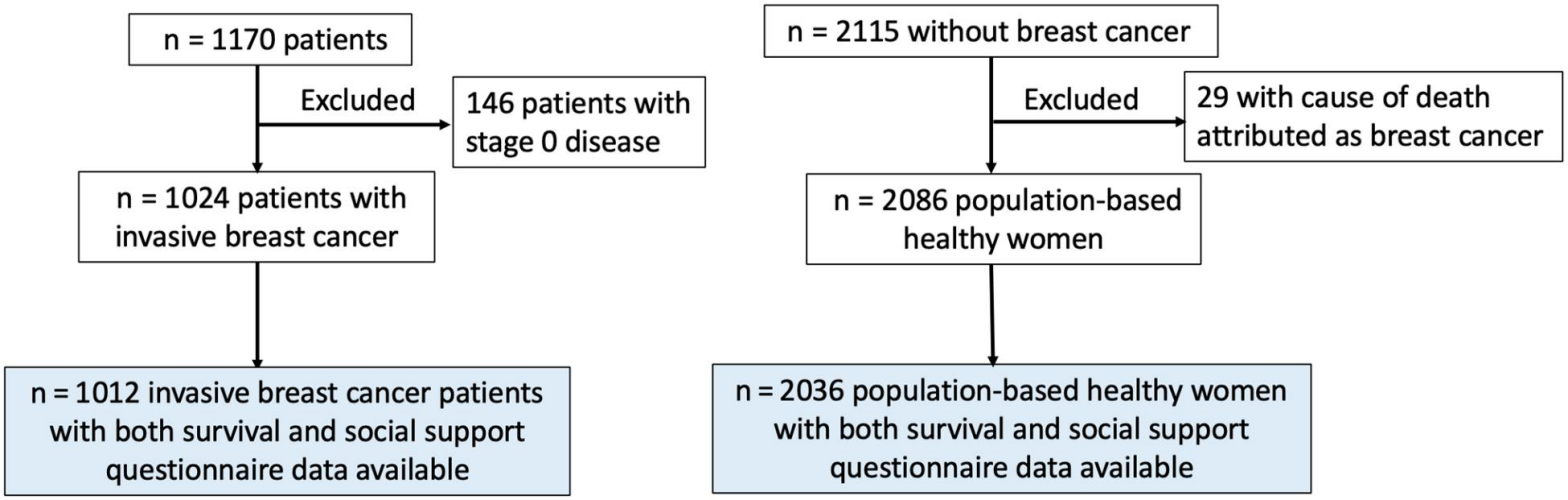
Flow diagram of patient selection schema.

The Western New York Exposures and Breast Cancer study is a prospective cohort study. It was originally a population-based case-control study in Erie and Niagara counties, conducted between 1996 and 2001 (start of follow-up). Study participants have been prospectively followed for mortality using the National Death Index (NDI). The study design and methods have been described in detail (16-18). Breast cancer patients (n = 1170) included women aged 35-79 years; residents of the 2 counties; diagnosed with primary, histologically confirmed, incident, invasive breast cancer; identified by nurse case finders in all the major hospitals in the study region (response rate of 72% among eligible cases). A population-based sample of healthy women (n = 2115), frequency matched on age with the breast cancer patients, was randomly selected from among residents of the 2 counties, using lists of licensed drivers for those aged 35-64 years and lists from the Health Care Financing Association for those aged 65-79 years (response rate of 65% among eligible healthy women). Healthy women with deaths attributed to breast cancer were excluded. Our analyses are limited to women with complete information on social support questionnaires and mortality (n = 1012 breast cancer patients, n = 2036 healthy women) (see Figure 1). Written informed consent was obtained from all the participants; the study was approved by the institutional review boards of the State University of New York, University at Buffalo, and all participating hospitals.

### Study measures

The following SDOH were queried at the Western New York Exposures and Breast Cancer study baseline interview: social support, marital status, household size, and income. Participants were asked 4 social support questions: 1) how many close friends they had (people they felt at ease with, could talk to about private matters, or could call on for help); 2) how many times they saw close friends or relatives during an average month (nearly daily, about once a week, about once a month, or less often); 3) did they have a confidante if they wanted to talk about something very personal (yes or no); 4) was there someone they could call on to help if they needed help, if the car broke down, or if they needed a ride somewhere (yes or no). Frequency of seeing friends was coded as 30 for daily, 4 for once a week, 1 for once a month, and 0.5 for less than once a month. Categories of low, medium, and high social support were obtained by multiplying number of friends by frequency of seeing them; the variable was then analyzed in tertiles. Marital status was reported as never married, married, widowed, divorced or separated, and living as if married and categorized as those with a spouse or partner (married, living with as if married) or without. Reported annual household income was categorized into less than $10 000, at least $10 000-$50 000, more than $50 000-$100 000, and more than $100 000. Household size was categorized as living alone, 2, or more than 2 members.

### Assessment of outcomes

Vital status was determined from NDI through December 31, 2018, using the *International Classification of Disease, Tenth Revision*, to systematically identify the cause of death. All-cause mortality was defined as death from any cause, and breast cancer–specific mortality was attributed if breast cancer was specifically identified as the cause of death.

### Covariate measurement

Data were collected by trained interviewers during in-person computer-assisted interviews at baseline on demographics, history of smoking, reproductive history, and physical activity. Height and weight were measured by trained interviewers using a standardized protocol. Clinical characteristics of tumors from women with breast cancer (stage, estrogen receptor status, and progesterone receptor status) were obtained from baseline medical charts reviewed by trained nurses. Comorbidity status was determined by participant report. We used a modified version of the Charlson Comorbidity index (19) for our study. Patients who answered yes to any of the following were categorized as having comorbidities present: myocardial infarction, peripheral vascular disease, cerebrovascular accident, transient ischemic attack, chronic obstructive pulmonary disease, liver cirrhosis, hepatitis, diabetes mellitus, or moderate to severe kidney disease. By study design, there were no participants eligible to participate with leukemia or lymphoma. Information on other components of the Charlson Comorbidity index, namely, congestive heart failure, dementia, connective tissue disease, peptic ulcer disease, hemiplegia, and AIDS, was not available. Data on high blood pressure and high blood cholesterol were collected and reported separately, as these are not included in the Charlson Comorbidity index score.

### Statistical analyses

Descriptive characteristics of those who died and those who remained alive, among breast cancer patients and healthy women, until the end of the follow-up were compared and compared among those with 0-1 or at least 2 friends, frequency of seeing them, and those with high vs low social support. *T* tests and analysis of variance were used to examine means and standard deviations of continuous normally distributed variables. For non-normal continuous variables, we present the median and interquartile ranges using the Wilcoxon rank sum test. Frequencies and percentages were reported for categorical variables, and χ^2^ test used for group comparisons. The *P* value for statistical significance (2-sided) of overall model effects was set at .05. Approximately 15% of subjects were missing data on stage or income. We compared participant characteristics for those with available or missing data. For stage, there were no statistically significant differences. For income, there were differences only for age; participants with income data were somewhat younger on average. Because the missingness is noninformative and should not bias any analyses, if a subject was missing a value for any variable being used in a given test or model, the subject was excluded from that specific test or model. Hazard ratios (HRs) and 95% confidence intervals (CIs) for total and breast cancer–specific mortality were estimated with multivariable Cox proportional hazards models among breast cancer patients and population-based healthy women separately. We also performed a combined analysis of breast cancer patients and healthy women, testing for interaction by group (patients or healthy women). We included covariates based on a priori knowledge of risk factors for breast cancer survival. We examined the inclusion of each model adjusting for other SDOH variables. Final models for the other SDOH included adjustment for income. Because deaths within the first year of diagnosis might reflect underlying illness and are unlikely related to social support measures, we carried out sensitivity analyses excluding women who died within 1 year of diagnosis (n = 3 for breast cancer, and n = 4 for healthy women). The results of these analyses were similar, and we present the hazard ratios from the complete sample.

## Results

Descriptive characteristics by all-cause mortality of breast cancer patients and healthy women are shown in Table 1. Total follow-up time for breast cancer patients from date of diagnosis ranged from 8.9 months to 21.9 years, with a median of 18.6 years, and for healthy women, follow-up time from the date of the interview ranged from 4.9 months to 22.7 years, with a median of 20.5 years. A total of 441 (43.6%) breast cancer patients and 660 (32.4%) healthy women died by the end of follow-up (December 31, 2018). Compared with women who survived to the end of the follow-up, those who died were on average older, postmenopausal, less educated, lower income, more likely to live alone, smoked more, less physically active, with higher body mass index, higher cancer stage, less likely to have received postmenopausal hormone therapy, and more likely to have high blood pressure, high blood cholesterol, and comorbidities.

**Table 1.** Descriptive characteristics of breast cancer patients and population-based healthy women by vital status

Covariates	Breast cancer patients				Population-based healthy women			
	Total (n = 1012)	Alive (n = 571)	Dead (n = 441)	*P* ^c^	Total (n = 2036)	Alive (n = 1376)	Dead (n = 660)	*P* ^c^
Age,^a^ mean (SD), y	58.0 (11.24)	54.6 (9.67)	62.5 (11.54)	**<.01**	57.5 (11.8)	53.1 (10.34)	66.7 (8.92)	**<.01**
Race,^b^ No. (%)								
Non-Hispanic White	928 (91.7)	531 (92.9)	397 (90.0)	.09	1854 (91.1)	1288 (93.6)	566 (85.8)	**<.01**
Education,^a^ mean (SD), y	13.5 (2.58)	13.9 (2.46)	12.9 (2.62)	**<.01**	13.4 (2.34)	13.7 (2.34)	12.5 (2.12)	**<.01**
Annual household income,^b^ No. (%)				**<.01**				**<.01**
<$10 000	73 (8.5)	22 (4.5)	51 (13.7)		122 (6.5)	37 (2.9)	85 (14.6)	
≥$10 000-$50 000	467 (54.4)	237 (48.9)	230 (61.7)		1075 (57.5)	664 (51.6)	411 (70.5)	
≥$50 001-$100 000	258 (30.1)	178 (36.7)	80 (21.5)		579 (30.9)	500 (38.9)	79 (13.6)	
>$100 000	60 (6.9)	48 (9.9)	12 (3.2)		93 (4.9)	85 (6.6)	8 (1.4)	
Household size,^b^ No. (%)				**<.01**				**<.01**
Living alone	244 (24.1)	93 (16.3)	151 (34.2)		438 (21.5)	204 (14.8)	234 (35.5)	
2	448 (44.3)	262 (45.9)	186 (42.2)		845 (41.5)	538 (39.1)	307 (46.5)	
>2	320 (31.6)	216 (37.8)	104 (23.6)		753 (36.9)	634 (46.1)	119 (18.0)	
Health insurance coverage,^b^ No. (%)				.47				.51
Yes	1005 (99.3)	568 (99.5)	437 (99.1)		2013 (98.9)	1359 (98.8)	654 (99.1)	
No	7 (0.7)	3 (0.5)	4 (0.9)		23 (1.1)	17 (1.2)	6 (0.9)	
Lifetime smoking,^d^ mean (SD), pack-years	0.9 (20.0)	0.2 (14.5)	3.0 (25.0)	**<.01**	0.2 (17.2)	0.0 (12.5)	2.6 (28.65)	**<.01**
Physical activity,^d^ mean (SD), metabolic equivalents in last 7 days	235.5 (14.38)	236.25 (15.75)	234.5 (14.4)	**<.01**	241.2 (24.25)	250.5 (132.75)	234.5 (19.25)	**<.01**
Body mass index,^a^ mean (SD), kg/m^2^	28.6 (6.43)	27.8 (6.15)	29.7 (6.64)	**<.01**	28.2 (6.32)	27.7 (5.95)	29.4 (6.88)	**<.01**
Age at first childbirth,^b^ No. (%)				.84				.55
Nulliparous	172 (17.0)	98 (17.2)	74 (16.8)		243 (11.9)	168 (12.2)	75 (11.4)	
Younger than 24 y	305 (30.1)	167 (29.3)	138 (31.3)		613 (30.1)	403 (29.3)	210 (31.8)	
24-27 y	296 (29.3)	166 (29.1)	130 (29.5)		659 (32.4)	443 (32.2)	216 (32.7)	
Older than 27 y	239 (23.6)	140 (24.5)	99 (22.5)		521 (25.6)	362 (26.3)	159 (24.1)	
Postmenopause,^b^ No. (%)	727 (71.8)	367 (64.3)	360 (81.6)	**<.01**	1432 (70.3)	814 (59.2)	618 (93.6)	**<.01**
Stage at diagnosis,^b^ No. (%)				**<.01**	—	—	—	
Stage I	480 (56.2)	309 (62.9)	171 (47.1)					
Stage II	314 (36.8)	165 (33.6)	149 (41.1)					
Stage III/IV	60 (7.0)	17 (3.5)	43 (11.9)					
Estrogen receptor status,^b^ No. (%)				.83	—	—	—	
Negative	279 (29.3)	160 (29.6)	119 (28.9)					
Positive	673 (70.7)	381 (70.4)	292 (71.1)					
Progesterone receptor status,^b^ No. (%)				.10	**—**	—	—	
Negative	352 (37.4)	187 (35.2)	165 (40.3)					
Positive	589 (62.6)	345 (64.9)	244 (59.7)					
Postmenopausal hormone therapy,^b^^,e^ No. (%)				**<.01**				**<.01**
Yes	376 (52.4)	226 (61.9)	150 (42.6)		693 (50.2)	454 (57.6)	239 (40.4)	
No	341 (47.6)	139 (38.1)	202 (57.4)		687 (49.8)	334 (42.4)	353 (59.6)	
High blood pressure,^b^ No. (%)				**<.01**				**<.01**
Yes	360 (35.8)	151 (26.6)	209 (47.6)		670 (33.0)	305 (22.2)	365 (55.6)	
No	646 (64.2)	416 (73.4)	230 (52.4)		1359 (67.0)	1067 (77.8)	292 (44.4)	
High blood cholesterol,^b^ No. (%)				**<.01**				**<.01**
Yes	349 (35.2)	172 (30.5)	177 (41.4)		720 (36.0)	431 (31.6)	289 (45.2)	
No	643 (64.8)	392 (69.5)	251 (58.6)		1282 (64.0)	931 (68.4)	351 (54.8)	
Comorbidity,^b^ No. (%)				**<.01**				**<.01**
Yes	175 (17.8)	62 (11.2)	113 (26.5)		321 (17.5)	112 (9.2)	209 (33.9)	
No	807 (82.2)	494 (88.9)	313 (73.5)		1516 (82.5)	1109 (90.8)	407 (66.1)	
Ever alcohol consumption,^b^ No. (%)				**<.01**				**<.01**
Nondrinkers	174 (17.3)	82 (14.5)	92 (20.9)		308 (15.2)	175 (12.8)	133 (20.3)	
Drinkers	833 (82.7)	485 (85.5)	348 (79.1)		1713 (84.8)	1190 (87.2)	523 (79.7)	
Marital status,^b^ No. (%)				**<.01**				**<.01**
Never married	84 (8.4)	51 (8.9)	33 (7.6)		107 (5.3)	74 (5.4)	33 (5.1)	
Married	608 (60.6)	382 (67.3)	226 (51.8)		1328 (65.6)	998 (72.8)	330 (50.5)	
Widowed	157 (15.6)	48 (8.5)	109 (25.0)		333 (16.4)	121 (8.8)	212 (32.4)	
Divorced or separated	136 (13.6)	74 (13.0)	62 (14.2)		224 (11.1)	151 (11.0)	73 (11.2)	
Live with as if married	19 (1.9)	13 (2.3)	6 (1.4)		33 (1.6)	27 (2.0)	6 (0.9)	

a Mean (SD) estimates were calculated for all continuous variables. “—” signifies breast cancer–specific variables, and hence, no values were available for healthy women. Bolded *P* values are <.05 and are statistically significant.

b Frequency and percentages were calculated for all categorical variables.

c *P* value compares breast cancer patients and healthy women who were alive and those who died; means were compared by *t* test and Wilcoxon rank sum test and frequencies by χ^2^ test or Fisher exact test.

d Median and interquartile range reported.

e Only for postmenopausal women.

The correlation between the number of close friends and the frequency of seeing them was weak (Spearman correlation coefficient = 0.14-0.16), and hence, we analyzed the 2 social support questions separately. Descriptive characteristics were compared for those with 0-1 and at least 2 close friends (Table 2). A total of 917 (90.6%) breast cancer patients, and a similar percentage (90.2%) of healthy women reported having at least 2 close friends. Compared with those with more friends, both breast cancer patients and healthy women with 0-1 friend were more likely to be older, postmenopausal, less educated, lower income, living alone, less likely to be married, and more likely to have comorbidities. We also examined the distribution of these characteristics by frequency of seeing friends and relatives and composite measure of social support (Supplementary Tables 1 and 2, available online). Similar, though weaker, associations as above were observed.

**Table 2.** Descriptive characteristics of breast cancer patients and population-based healthy women by number of close friends

Covariate	Breast cancer patients			Population-based healthy women		
	Close friends 0-1 (n = 95)	Close friends ≥2 (n = 917)	*P* ^c^	Close friends 0-1 (n = 199)	Close friends ≥2 (n = 1837)	*P* ^c^
Age,^a^ mean (SD), y	62.1 (11.50)	57.6 (11.13)	**<.01**	59.7 (11.54)	57.3 (11.76)	**<.01**
Race,^b^ No. (%)						
Non-Hispanic White	80 (84.2)	848 (92.5)	**<.01**	172 (86.4)	1682 (91.6)	**.02**
Education,^a^ mean (SD), y	12.5 (2.66)	13.6 (2.55)	**<.01**	13.0 (2.16)	13.4 (2.35)	**.03**
Income, No. (%)			**<.01**			**<.01**
<$10 000	17 (21.3)	56 (7.2)		19 (10.8)	103 (6.1)	
≥$10 000-$50 000	46 (57.5)	421 (54.1)		112 (63.6)	963 (56.9)	
≥$50 001-$100 000	13 (16.3)	245 (31.5)		38 (21.6)	541 (31.9)	
>$100 000	4 (5)	56 (7.2)		7 (4.0)	86 (5.1)	
Household size, No. (%)			**<.01**			**.02**
Living alone	43 (45.3)	201 (21.9)		57 (28.6)	381 (20.7)	
2	28 (29.5)	420 (45.8)		82 (41.2)	763 (41.5)	
>2	24 (25.3)	296 (32.3)		60 (30.2)	693 (37.7)	
Health insurance, No. (%)			.08			.59
Yes	93 (97.9)	912 (99.5)		197 (98.5)	1817 (98.9)	
No	2 (2.1)	5 (0.6)		3 (1.5)	20 (1.1)	
Lifetime smoking,^d^ mean (SD), pack-years	3.2 (32.1)	0.7 (17.4)	**.04**	1.0 (20.0)	0.2 (17.0)	.28
Physical activity,^d^ mean (SD), metabolic equivalents in last 7 days	234.0 (15.75)	235.8 (14.25)	.27	239.9 (23.00)	241.5 (24.00)	.13
Body mass index,^a^ mean (SD), kg/m^2^	28.8 (6.15)	28.6 (6.46)	.80	28.9 (6.32)	28.1 (6.31)	.10
Postmenopausal,^b^ No. (%)	79 (83.2)	648 (70.7)	**.01**	153 (76.9)	1279 (69.6)	**.03**
Stage at diagnosis,^b^ No. (%)			**<.01**	—	—	—
Stage I	50 (62.5)	430 (55.6)				
Stage II	26 (32.5)	288 (37.2)				
Stage III/IV	4 (5)	56 (7.2)				
Estrogen receptor status,^b^ No. (%)			.96	—	—	—
Negative	26 (29.6)	253 (29.3)				
Positive	62 (70.5)	611 (70.7)				
Progesterone receptor status,^b^ No. (%)			.63	—	—	—
Negative	35 (39.8)	317 (37.2)				
Positive	53 (60.2)	536 (62.8)				
Postmenopausal hormone therapy,^b^^,^^e^ No. (%)			.89			.63
Yes	42 (53.2)	334 (52.4)		73 (48.3)	620 (50.5)	
No	37 (46.8)	304 (47.7)		78 (51.7)	609 (49.6)	
High blood pressure,^b^ No. (%)			**<.01**			.82
Yes	51 (54.3)	309 (33.9)		64 (32.3)	606 (33.1)	
No	43 (45.7)	603 (66.1)		134 (67.7)	1225 (66.9)	
High blood cholesterol,^b^ No. (%)			**.03**			.15
Yes	42 (45.1)	307 (34.2)		80 (40.6)	640 (35.5)	
No	51 (54.8)	592 (65.9)		117 (59.4)	1165 (64.5)	
Comorbidity,^b^ No. (%)			.15			**.01**
Yes	21 (23.3)	154 (17.3)		41 (24.3)	280 (16.8)	
No	69 (76.7)	738 (82.7)		128 (75.7)	1388 (83.2)	
Ever alcohol consumption,^b^ No. (%)			.43			.70
Nondrinkers in lifetime	19 (20.2)	155 (17.0)		32 (16.2)	276 (15.1)	
Drinkers in lifetime	75 (79.8)	758 (83.0)		166 (83.8)	1547 (84.9)	
Marital status,^b^ No. (%)			**<.01**			**<.01**
Never married	13 (13.9)	71 (7.8)		19 (9.6)	88 (4.8)	
Married	41 (44.1)	567 (62.2)		116 (58.9)	1212 (66.3)	
Widowed	18 (19.4)	139 (15.3)		34 (17.3)	299 (16.4)	
Divorced or separated	19 (20.4)	117 (12.8)		26 (13.2)	198 (10.8)	
Live with as if married	2 (2.2)	17 (1.9)		2 (1.0)	31 (1.7)	

a Mean (SD) estimates were calculated for all continuous variables. “—” signifies breast cancer–specific variables, and hence, no values were available for healthy women. Bolded *P* values are <.05 and are statistically significant.

b Frequency and percentages were calculated for all categorical variables.

c *P* values compare breast cancer patients and healthy women by 0-1 and more than 2 close friends, means were compared by *t* test and Wilcoxon rank sum test and frequencies by χ^2^ test or Fisher exact test.

d Median and interquartile range.

e Only for postmenopausal patients.

**Figure 2.**
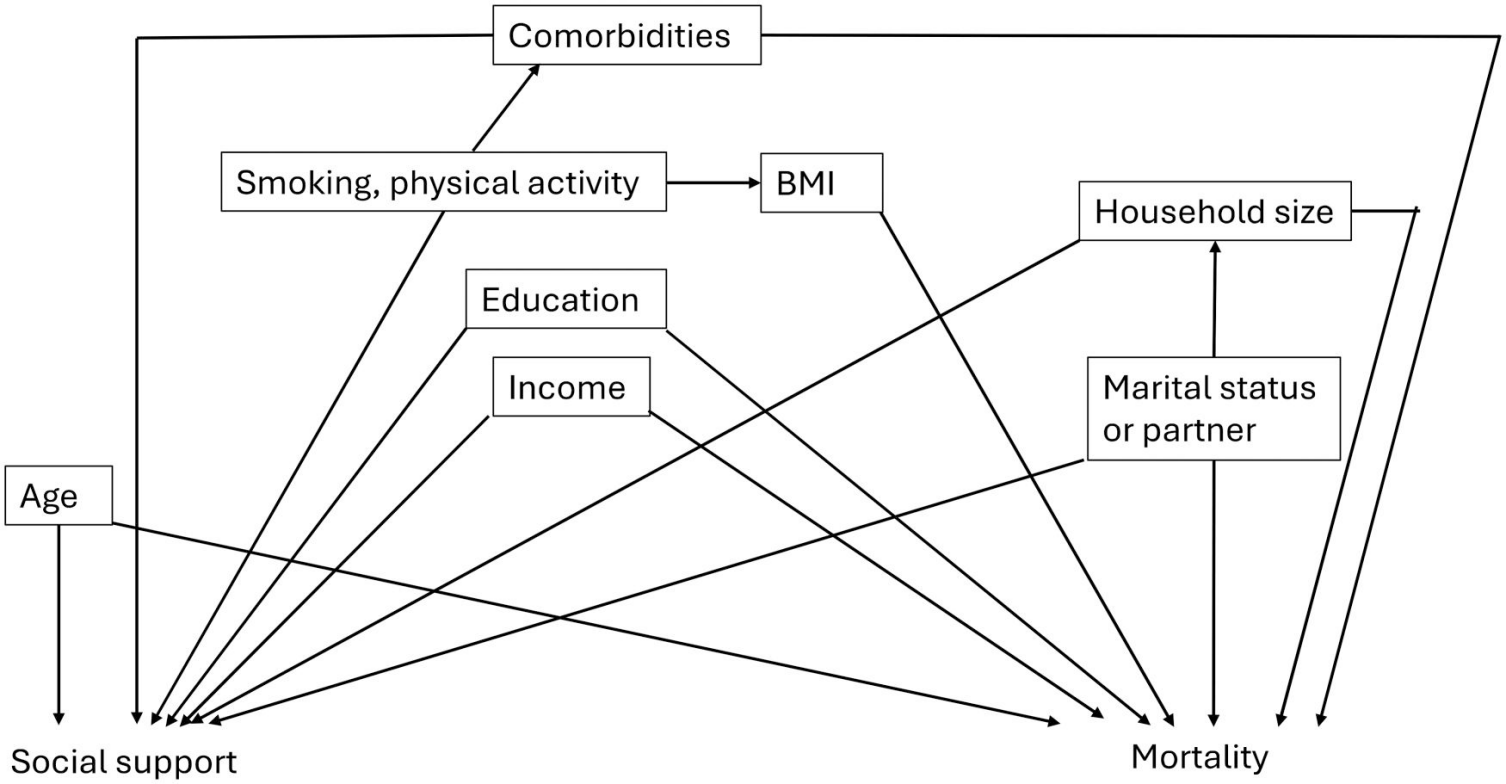
Directed acyclic graph showing conceptual framework for the study assessing social support and mortality with accompanying covariates. BMI = body mass index.

Next, we examined the association of number of friends, frequency of seeing them, and composite measure of social support with all-cause and breast cancer–specific mortality. We considered age, smoking, physical activity, body mass index, education, income, high blood pressure, high cholesterol, comorbidities, and cancer stage (breast cancer patients only) as potential covariates (Figure 2 shows directed acyclic graph); age and income were the only variables that affected point estimates and were therefore adjusted for in our final models. Given the a priori poor survival associated with higher stage and smoking, these were also included. Compared with those with at least 2 friends, having 0-1 friend was not associated with all-cause (HR = 1.21, 95% CI = 0.85 to 1.73) or breast cancer–specific mortality (HR = 1.37, 95% CI = 0.76 to 2.45). Similarly, there was no association with mortality observed among healthy women (HR = 1.11, 95% CI = 0.86 to 1.43) (Table 3). Compared with seeing friends or relatives daily, having less frequent contacts was not associated with all-cause mortality (HR = 0.71, 95% CI = 0.46 to 1.10; *P*_trend_ = .08) for breast cancer patients or healthy women (Supplementary Table 3, available online). Similarly, there was no association of composite measure of social support with mortality (Supplementary Table 4, available online).

**Table 3.** Number of close friends and mortality among breast cancer patients and population-based healthy women, multivariable adjusted hazard ratios and 95% confidence intervals

Social support	Breast cancer patients		Population-based healthy women	
	HR (95% CI)	HR (95% CI)	HR (95% CI)	HR (95% CI)
Social support (Number of close friends)	≥2 (n = 917)	0-1 (n = 95)	≥2 (n = 1837)	0-1 (n = 199)
Total mortality, No.	385	56	580	80
Age adjusted^a^	1.0 (Referent)	1.30 (0.98 to 1.73)	1.0 (Referent)	1.12 (0.88 to 1.41)
Age and income adjusted^b^	1.0 (Referent)	1.19 (0.87 to 1.62)	1.0 (Referent)	1.09 (0.85 to 1.41)
Multivariable adjusted^c^	1.0 (Referent)	1.21 (0.85 to 1.73)	1.0 (Referent)	1.11 (0.86 to 1.43)
Breast cancer–specific mortality, No.	151	17	—	—
Age adjusted^a^	1.0 (Referent)	1.24 (0.75 to 2.05)	—	—
Age and income adjusted^b^	1.0 (Referent)	1.14 (0.66 to 1.96)		
Multivariable adjusted^c^	1.0 (Referent)	1.37 (0.76 to 2.45)		

a n = 1012 for breast cancer patients and n = 2036 for population-based healthy women. “—” signifies breast cancer–specific variables, and hence, no values were available for healthy women. CI = confidence interval; HR = hazard ratio.

b Age and income-adjusted: age (years), income (<$10 000, $10 000-$50 000, $100 000, >$100 000 [referent]); n = 858 for breast cancer patients, and n = 1869 for population-based healthy women.

c Multivariable-adjusted: age (years), smoking (life pack-years), income (<$10 000, $10 000-$50 000, $50 001-$100 000, >$100 000 [referent]). Only for breast cancer patients, cancer stage (stage I [the referent group], stage II, stage III/IV) was included in the model; n = 731 for breast cancer patients, and n = 1867 for population-based healthy women.

Associations of other SDOH, income, marital status, and household size with mortality were also examined (Tables 4 and 5). Compared with those with annual household income of more than $100 000, breast cancer patients with lower income (<$10 000) had higher all-cause (HR = 2.48, 95% CI = 1.24 to 4.97; *P_trend_* *<* .01) and a nonsignificant increase in breast cancer–specific mortality (HR = 1.83, 95% CI = 0.71 to 4.73). Among healthy women, there was increased all-cause mortality associated with lower income (HR = 2.63, 95% CI = 1.25 to 5.53; *P_trend_* *<* .01) (Table 4). Neither not having a spouse or partner nor household size was associated with mortality in either group (Table 5).

**Table 4.** Income and mortality among breast cancer patients and population-based healthy women, multivariable adjusted hazard ratios and 95% confidence intervals

Income	Total mortality, No.	Age adjusted,^a^HR (95% CI)	Multivariable adjusted,^b^HR (95% CI)	Breast cancer–specific mortality, No.	Age adjusted,^a^HR (95% CI)	Multivariable adjusted,^b^HR (95% CI)
Breast cancer patients						
>$100 000 (n = 60)	12	1.0 (Referent)	1.0 (Referent)	9	1.0 (Referent)	1.0 (Referent)
>$50 000-$100 000 (n = 258)	80	1.43 (0.78 to 2.63)	1.30 (0.69 to 2.46)	43	1.20 (0.58 to 2.46)	1.20 (0.56 to 2.57)
≥$10 000-$50 000 (n = 467)	230	2.03 (1.13 to 3.66)	1.81 (0.98 to 3.36)	84	1.64 (0.81 to 3.29)	1.49 (0.71 to 3.13)
<$10 000 (n = 73)	51	**3.55 (1.87 to 6.73)**	**2.48 (1.24 to 4.97)**	14	2.00 (0.86 to 4.68)	1.83 (0.71 to 4.73)
*P*_trend_		**<.01**	**<.01**		**.03**	.11
Population-based healthy women						
>$100 000 (n = 93)	8	1.0 (Referent)	1.0 (Referent)	**—**	**—**	**—**
>$50 000-$100 000 (n = 579)	79	1.49 (0.72 to 3.09)	1.44 (0.70 to 2.98)	**—**	**—**	**—**
≥$10 000-$50 000 (n = 1075)	411	2.11 (1.04 to 4.29)	1.92 (0.94 to 3.90)	**—**	**—**	**—**
<$10 000 (n = 122)	85	**3.10 (1.48 to 6.51)**	**2.63 (1.25 to 5.53)**	**—**	**—**	**—**
*P*_trend_		**<.01**	**<.01**	**—**	**—**	**—**

a n = 858 for breast cancer patients, and n = 1869 for population-based healthy women. “—” signifies breast cancer–specific variables, and hence, no values were available for healthy women. Bolded *P* values are statistically significant as <.05. CI = confidence interval; HR = hazard ratio.

b Multivariable-adjusted: age (years), smoking (lifetime pack-years). Only for breast cancer patients, cancer stage (stage I [the referent group], stage II, stage III/IV) was included in the model; n = 731 for breast cancer patients, and n = 1867 for population-based healthy women.

**Table 5.** Marital status and household size and mortality among breast cancer patients and population-based healthy women, multivariable adjusted hazard ratios and 95% confidence intervals

Marital status and household size	Total mortality, No.	Age-adjusted, ^a^ HR (95% CI)	Age and income-adjusted, ^b^ HR (95% CI)	Multivariable adjusted, ^c^ HR (95% CI)	Breast cancer–specific mortality, No.	Age-adjusted, ^a^ HR (95% CI)	Age and income-adjusted, ^b^ HR (95% CI)	Multivariable adjusted, ^c^ HR (95% CI)
Breast cancer patients								
Marital status								
Married or living as if married (n = 627)	232	1.0 (Referent)	1.0 (Referent)	1.0 (Referent)	110	1.0 (Referent)	1.0 (Referent)	1.0 (Referent)
Never married or widowed or divorced or separated (n = 377)	204	**1.41 (1.17 to 1.72)**	1.09 (0.87 to 1.37)	1.00 (0.78 to 1.29)	57	1.00 (0.73 to 1.39)	0.76 (0.52 to 1.11)	0.73 (0.49 to 1.09)
Population-based healthy women								
Marital status								
Married or living as if married (n = 1361)	336	1.0 (Referent)	1.0 (Referent)	1.0 (Referent)	**—**	**—**	**—**	**—**
Never married or widowed or divorced or separated (n = 664)	318	**1.35 (1.15 to 1.58)**	1.19 (0.99 to 1.43)	1.14 (0.95 to 1.37)	**—**	**—**	**—**	**—**
Breast cancer patients								
Household size								
>2 (n = 320)	104	1.0 (Referent)	1.0 (Referent)	1.0 (Referent)	62	1.0 (Referent)	1.0 (Referent)	1.0 (Referent)
2 (n = 448)	186	0.78 (0.60 to 1.01)	0.91 (0.68 to 1.20)	0.96 (0.71 to 1.32)	68	0.95 (0.65 to 1.39)	1.09 (0.73 to 1.63)	1.27 (0.83 to 1.95)
Living alone (n = 244)	151	1.19 (0.90 to 1.58)	1.09 (0.79 to 1.49)	1.02 (0.71 to 1.45)	38	1.14 (0.73 to 1.79)	1.05 (0.64 to 1.72)	1.28 (0.75 to 2.21)
*P*_trend_		.07	.50	.89		.60	.81	.32
Population-based healthy women								
Household size								
>2 (n = 753)	119	1.0 (Referent)	1.0 (Referent)	1.0 (Referent)	**—**	**—**	**—**	**—**
2 (n = 845)	307	0.99 (0.80 to 1.25)	1.04 (0.82 to 1.33)	1.00 (0.80 to 1.28)	**—**	**—**	**—**	**—**
Living alone (n= 438)	234	1.21 (0.95 to 1.54)	1.16 (0.89 to 1.51)	1.05 (0.80 to 1.40)	**—**	**—**	**—**	**—**
*P_t_*_rend_		.07	.24	.70	—	—	—	—

a Age-adjusted: n = 1012 for breast cancer patients, n = 2036 for population-based healthy women (for association of household size and mortality); n = 1002 for breast cancer patients, n = 2025 for population-based healthy women (for association of marital status and mortality). ^“—”^ signifies breast cancer–specific variables, and hence, no values were available for healthy women. CI = confidence interval; HR = hazard ratio.

b Age and income-adjusted: age (years), income (<$10 000, $10 000-$50 000, $50 001-$100 000, >$100 000 [Referent]), n = 858 for breast cancer patients, n = 1869 for population-based healthy women (for association of household size and mortality); n = 853 for breast cancer patients, n = 1860 for population-based healthy women (for association of marital status and mortality).

c Multivariable-adjusted: age (years), smoking (life pack-years), income (<$10 000, $10 000-$50 000, $50 001-$100 000, >$100 000 [Referent]). Only for breast cancer patients, cancer stage (stage I [the referent group], stage II, stage III/IV) was included in the mode; n = 731 for breast cancer patients, n = 1869 for population-based healthy women (for assessment of household size and mortality); n = 727 for breast cancer patients, n = 1858 for population-based healthy women (for association of marital status and mortality).

We tested the interaction of group (patients and healthy women) with social support or SDOH on survival. We observed an interaction in the association between household size and mortality, but it was not clinically relevant. There was no difference by group for the other variables.

## Discussion

In this population-based prospective study, mortality was higher among lower-income women, both those with a breast cancer diagnosis and those without. There was a suggestion of higher all-cause and breast cancer–specific mortality associated with fewer friends for both groups, but the confidence intervals included the null. There was weak evidence of lower all-cause mortality among breast cancer patients with lower frequency of contacts; again, the confidence intervals included the null.

Our observation that lower income was associated with higher all-cause mortality is similar to findings in another study of patients with breast cancer (20,21). Women with higher income were able to afford better cancer care (ie, access to clinical facilities, insurance, earlier diagnosis, and timely treatment). Given Surveillance, Epidemiology, and End Results data limitations, this study did not consider confounding factors, such as stage, which were included in our study. In another study, low income was associated with more aggressive breast cancer biology and worse survival (22). Low income could lead to less screening, consequent delay in diagnosis, and tumor biology with poorer prognosis (23). Financial distress can result in increased psychological and emotional distress, poor quality of life, depression (24), dysregulation in stress pathways (25), reduced tumor suppressor p53 function (26), and aggressive tumor biology with distant metastasis (27). In another study, women with lower household income were 1.44 times more likely to die from breast cancer compared with those with higher income, but adjustment for factors including screening mammography and stage eliminated disparity (28).

In our study, women with small social networks, defined by having 0-1 friend, were more likely to have adverse lifestyle factors, including older age, less education, lower income, living alone, smokers, and more comorbidities. These findings are similar to another study finding that socially isolated women were more likely to be obese, have low physical activity, be current smokers, and have higher alcohol intake compared with socially integrated women (29). Interestingly, when we studied associations of lifestyle factors with frequency of contacts, associations were weak, suggesting that number of friends may be a more meaningful indicator. Although the confidence interval included the null, we observed a trend toward worse survival with fewer friends. Our findings are similar to other studies showing that having no friends (12,13) or fewer friends (15) associated with an elevated risk or trend toward higher all-cause and breast cancer–specific mortality. Although some of these studies did not adjust for comorbidities or income, comorbidity adjustment in our study did not make any meaningful change (data not shown), but association was weaker with income adjustment.

Although we did not observe an association of frequency of contacts with mortality, we observed that fewer contacts were associated with a trend toward lower all-cause mortality among breast cancer patients, whereas no association was observed among healthy women. The former was contrary to our hypothesized association. It might be explained by lower social burden (caregiving responsibilities) associated with less frequent contacts at the time of breast cancer diagnosis, perhaps allowing the women to take care of themselves, manage side effects of treatment, or other health practices. These findings are similar to another study where women with large social networks but greater social burden were at increased risk of breast cancer–specific mortality (0-3 relatives [Referent]; 4-5 relatives: HR = 1.47, 95% CI = 0.62 to 3.52; 6-9 relatives: HR = 2.08, 95% CI = 0.89 to 4.86; ≥10 relatives: HR = 3.55, 95% CI = 1.35 to 9.33) (30). Also, it could be that those who need more caregiving are the ones who meet more frequently with their friends and relatives.

We examined associations of other SDOH related to social support, marital status, and household size with mortality. In contrast to other studies in which living alone (31) is associated with increased all-cause and breast cancer–specific mortality, we saw no such association. We also did not observe any association of marital status with mortality. Similar findings were observed (12) in other studies with breast cancer patients. However, some other studies have shown lower all-cause and breast cancer–specific mortality with marital status (32,33). Having a partner is associated with healthier lifestyles and better access to emotional and financial support. Some of these prior studies did not adjust for comorbidities and smoking, which may impact survival and were considered in our study.

In interpretating these findings, the strengths and weaknesses of the study should be considered. Strengths include use of a population-based study with long follow-up and inclusion of a comparison group of healthy women to examine if observed associations were specific to those with breast cancer or would be expected regardless of a breast cancer diagnosis. Limitations include use of the NDI to ascertain cause of death, particularly for the analyses of breast cancer–specific mortality (34). Residual confounding cannot be entirely ruled out, for example, absence of data on treatment administration and adherence (35); use of other sources of social support and transportation; other relevant social measures, such as Medical Outcomes Study questionnaire (36), community memberships; number of children and living parents; social burden, and social strain (37). There might be differences in recall of social support and other SDOH by women with and without breast cancer, resulting in misclassification, though likely that error was not differential by mortality status. The population included was predominantly White women (91.7%), and lack of racial diversity may limit external validity. There were some missing data for income and stage, which was noninformative and should not bias the general conclusions. Several variables, such as social support, marital status, income, and household size, are dynamic and may vary over time. Time-dependent changes in these variables were not known; results were based on a single baseline questionnaire. In one study (12), the level of social networks did not change markedly before and following diagnosis, highlighting that these dynamic changes may be minimal and, thus, not impact outcomes. Given the long follow-up, although we were interested in breast cancer deaths, there could be competing risks from non–breast cancer deaths, detailed data for which were not available given the limitations of NDI.

Moving forward, there is a need to design a questionnaire to comprehensively examine and collate different indices such as emotional support, social burden, social strain, and SDOH as they could modify the relationship of social support with mortality. Prospective intervention studies could be designed for patients in need of social support, such as counseling services, and financial assistance programs for those with lower income. Given the increased mortality risk associated with lower income, additional studies to understand the sources of that difference and identify the most effective interventions are needed. The performance of these interventions could be analyzed by longitudinal monitoring of questionnaire data and other objective biomarker measures such as epinephrine, norepinephrine, and cortisol to measure psychosocial stress associated with lack of social support.

In this population-based study, we observed that lower income was associated with poorer survival, with a similar magnitude of association for women diagnosed with breast cancer and healthy women. Neither the number nor the frequency of contact with friends was associated with mortality. A greater understanding of clinical outcome differences related to income as an important SDOH is needed. More studies to understand the impact of social contacts on cancer mortality are urgently needed given the surgeon general advisory considering “loneliness and isolation” as a “public health crisis.” Future studies should consider incorporating assessment of financial resources with outpatient clinic visits and designing prospective intervention studies to improve survival for those with fewer resources.

## Supplementary Material

pkae057_Supplementary_Data

## Data Availability

All data used in this article are available in WEB study. Data analyzed in this study are included in this published article and its appendix. The de-identified data for these analyses can be shared on the basis of reasonable requests to the corresponding author upon approval by the WEB study research committee.
