# Supplementary material for: Social networks, social determinants, and mortality: Western New York Exposures and Breast Cancer study
Source: JNCI Cancer Spectr. 2024 Jul 17;8(4):pkae057. doi: 10.1093/jncics/pkae057 (PMC11288187; doi:10.1093/jncics/pkae057)
Supplement: pkae057_Supplementary_Data [file pkae057_supplementary_data.docx]

**Supplementary Table 1.** Descriptive characteristics of breast cancer cases and population-based healthy women by frequency of seeing close friends or relatives.

|  | **Breast cancer cases** | | | | **Population-based healthy women** | | | |
| --- | --- | --- | --- | --- | --- | --- | --- | --- |
|  | **How many times do you see your close friends or relatives** | | | | | | | |
| **Covariate** | **Daily**  **(n=584)** | **Once a week**  **(n=341)** | **Once a month or less often (n=87)** | **p^c^** | **Daily**  **(N=955)** | **Once a week**  **(N=804)** | **Once a month or less often**  **(N=277)** | **p^c^** |
| Age (years)^a^ | 57.4+/-0.46 | 59.0+/- 0.61 | 58.3+/-1.20 | 0.09 | 57.6+/-0.38 | 57.1+/-0.41 | 58.6+/-0.71 | 0.19 |
| Race (N, %)  Non-Hispanic White ^b^ | 529, 90.6% | 322, 94.4% | 77, 88.5% | 0.07 | 865, 90.6% | 737, 91.7% | 252, 90.9% | 0.73 |
| Education (years) ^a^ | 13.5+/-0.11 | 13.4+/-0.14 | 13.4+/-0.28 | 0.56 | 13.4+/-0.08 | 13.3+/-0.08 | 13.3+/-0.14 | 0.54 |
| Income ($) ^b^  <$10,000  ≥$10,000 – 50,000  >$50,001 – 100,000  >$100,000 | 35, 7.4%  253, 53.4%  143, 30.2%  43, 9.1% | 27, 8.9%  173, 56.9%  91, 29.9%  13, 4.3% | 11, 13.8%  41, 51.3%  24, 30%  4, 5% | **<0.01** | 54, 6.2%  494, 57.1%  281, 32.5%  36, 4.2% | 43, 5.7%  438, 58.2%  227, 30.2%  44, 5.9% | 25, 9.9%  143, 56.8%  71, 28.2%  13, 5.2% | 0.16 |
| Household size ^b^  Living alone  2  >2 | 134, 23.0%  253, 43.3%  197, 33.7% | 85, 24.9%  156, 45.8%  100, 29.3% | 25, 28.7%  39, 44.8%  23, 26.4% | 0.46 | 204, 21.4%  402, 42.1%  349, 36.5% | 175, 21.8%  320, 39.8%  309, 38.4% | 59, 21.3%  123, 44.4%  95, 34.3% | 0.68 |
| Health insurance ^b^  Yes  No | 578, 98.9%  6, 1.0% | 341, 100%  0 | 86, 98.9%  1, 1.2% | **0.02** | 943, 98.7%  12, 1.3% | 797, 99.1%  7, 0.9% | 273, 98.6%  4, 1.4% | **0.03** |
| Lifetime smoking ^d^  (pack-years) | 0.6 +/- 18.0 | 1.1 +/- 21.5 | 0.2 +/- 18.25 | 0.38 | 0.1 +/- 17.0 | 0.1 +/- 15.2 | 1.7 +/- 21.4 | 0.19 |
| Physical activity ^d^  (METs in last 7 days) | 235.0+/-13.5 | 235.8+/-16.25 | 235.5 +/- 16.25 | 0.30 | 241.0+/-23.75 | 242.2+/-24.0 | 239.7+/-24.75 | 0.63 |
| BMI (kg/m^2^) ^a^ | 28.7+/-0.27 | 28.6+/-0.35 | 28.3+/-0.69 | 0.88 | 28.4+/-0.20 | 27.8+/-0.22 | 28.6+/-0.38 | 0.07 |
| Postmenopausal (n, %) ^b^ | 405, 69.4% | 259, 75.9% | 63, 72.4% | 0.10 | 667, 69.8% | 559, 69.5% | 206, 74.4% | 0.28 |
| Stage at diagnosis (n, %) ^b^  Stage I  Stage II  Stage III/ IV | 269, 53.9%  192, 38.5%  38, 7.6% | 170, 59.4%  99, 34.6%  17, 5.9% | 41, 59.4%  23, 33.3%  5, 7.3% | **<0.01** |  |  |  |  |
| ER status (n, %) ^b^  Negative  Positive | 165, 29.8%  388, 70.2% | 93, 29.2%  226, 70.9% | 21, 26.3%  59, 73.8% | 0.80 | - | - | - |  |
| PR status (n, %) ^b^  Negative  Positive | 206, 37.7%  340, 62.3% | 112, 35.4%  204, 64.6% | 34, 43.0%  45, 57.0% | 0.45 | - | - | - |  |
| Postmenopausal hormone therapy (n, %) ^b,e^  Yes  No | 211, 52.5%  191, 47.5% | 131, 51.8%  122, 48.2% | 34, 54.8%  28, 45.2% | 0.91 | 325, 50.0%  325, 50.0% | 275, 51.6%  258, 48.4% | 93, 47.2%  104, 52.8% | 0.57 |
| High blood pressure (n, %) ^b^  Yes  No | 212, 36.5%  369, 63.5% | 116, 34.3%  222, 65.7% | 32, 36.8%  55, 63.2% | 0.79 | 321, 33.8%  630, 66.3% | 246, 30.7%  555, 69.3% | 103, 37.2%  174, 62.8% | 0.11 |
| High blood cholesterol (n, %) ^b^  Yes  No | 186, 32.5%  387, 67.5% | 133, 39.7%  202, 60.3% | 30, 35.7%  54, 64.3% | 0.09 | 339, 36.2%  598, 63.8% | 279, 35.1%  515, 64.9% | 102, 37.6%  169, 62.4% | 0.75 |
| Comorbidity ^b^  Yes  No | 107, 18.8%  461, 81.2% | 51, 15.5%  278, 84.5% | 17, 20.0%  68, 80.0% | 0.39 | 158, 18.1%  716, 81.9% | 115, 16.1%  598, 83.9% | 48, 19.2%  202, 80.8% | 0.44 |
| Ever alcohol consumption (n, %) ^b^  Non-drinkers in lifetime  Drinkers in lifetime | 108, 18.6%  474, 81.4% | 53, 15.7%  285, 84.3% | 13, 14.9%  74, 85.1% | 0.45 | 133, 14.0%  815, 85.9% | 121, 15.2%  677, 84.8% | 54, 19.6%  221, 80.4% | 0.08 |
| Marital status (n, %) ^b^  Never married  Married  Widowed  Divorced/ separated  Live with as if married | 51, 8.8%  348, 60.1%  87, 15.0%  81, 13.9%  12, 2.1% | 24, 7.1%  212, 62.7%  56, 16.6%  42, 12.4%  4, 1.2% | 9, 10.3%  48, 55.2%  14, 16.1%  13, 14.9%  3, 3.5% | **<0.01** | 51, 5.4%  614, 64.7%  154, 16.2%  115, 12.1%  15, 1.6% | 41, 5.1%  529, 66.2%  130, 16.3%  85, 10.6%  14, 1.8% | 15, 5.4%  185, 66.8%  49, 17.7%  24, 8.7%  4, 1.4% | **<0.01** |
| ^a^ Mean +/- standard error (SE) estimates were calculated for all continuous variables.  ^b^ Frequency and percentages were calculated for all categorical variables.  ^c^ p-value compares breast cancer cases and healthy women by frequency of seeing close friends, means by ANOVA and frequencies by chi-square test.  ^d^ Median and interquartile range  ^e^ only for postmenopausal patients.  -These are breast cancer specific variables and hence, no values available for healthy women | | | | | | | | |

**Supplementary Table 2.** Descriptive characteristics of breast cancer cases and population-based healthy women by composite social support levels

|  | **Breast cancer cases** | | | **p^c^** | **Population-based healthy women** | | | **p^c^** |
| --- | --- | --- | --- | --- | --- | --- | --- | --- |
| **Covariates** | **Low social support ^d^**  **(N=266)** | **Medium social support ^d^**  **(N=361)** | **High social support ^d^**  **(N=385)** |  | **Low social support ^d^**  **(N=739)** | **Medium social support ^d^**  **(N=693)** | **High social support ^d^**  **(N=604)** |  |
| Age (years)^a^ | 59.8 +/- 11.12 | 58.8 +/- 11.30 | 56.1 +/- 10.9 | **<0.01** | 57.7 +/- 11.4 | 58.0 +/- 12.0 | 56.8 +/- 11.83 | 0.19 |
| White (n, %) ^b^ | 240, 90.2% | 331, 91.7% | 357, 92.7% | 0.52 | 672, 90.9% | 621, 89.6% | 561, 92.9% | 0.12 |
| Education (years)^a^ | 13.2 +/- 2.33 | 13.2 +/- 2.48 | 13.9 +/- 2.78 | **<0.01** | 13.3+/-2.36 | 13.2 +/- 2.21 | 13.6 +/- 2.44 | **0.03** |
| Income ($) ^b^  <$10,000  $10,000-50,000  $50,001-100,000  >$100,000 | 29, 12.4%  135, 57.7%  60, 25.6%  10, 4.3% | 28, 9.3%  164, 54.5%  94, 31.2%  15, 5.0% | 16, 5.0%  168, 52.0%  104, 32.2%  35, 10.8% | **<0.01** | 56, 8.2%  388, 56.9%  198, 29.1%  39, 5.7% | 33, 5.2%  386, 60.8%  191, 30.1%  25, 3.9% | 33, 5.9%  301, 54.4%  190, 34.4%  29, 5.2% | 0.05 |
| Household size (n) ^b^  Living alone  2  >2 | 78, 29.3%  117, 44.0%%  71, 26.7% | 81, 22.4%  167, 46.3%  113, 31.3% | 85, 22.1%  164, 42.6%  136, 35.3% | 0.08 | 168, 22.7%  303, 41.0%  268, 36.3% | 149, 21.5%  287, 41.4%  257, 37.1% | 121, 20.0%  255, 42.2%  228, 37.8% | 0.84 |
| Health insurance ^b^  Yes  No | 265, 99,6%  1, 0.4% | 358, 99.2%  3, 0.8% | 382, 99.2%  3, 0.8% | 0.77 | 730, 98.8%  9, 1.2% | 686, 98.9%  7, 1.0% | 597, 98.8%  7, 1.2% | 0.93 |
| Lifetime smoking ^e^  (pack-years) | 2.3 +/- 23.0 | 0.3 +/- 18.0 | 0.9 +/- 17.2 | 0.31 | 0.6 +/- 19.0 | 0 +/- 14.2 | 0.3 +/- 17.0 | 0.40 |
| Physical activity ^e^  (METs in last 7 days) | 235.3 +/- 16.0 | 235.3 +/- 14.25 | 236.0 +/- 13.75 | 0.26 | 241.8+/-26.25 | 240.8 +/- 21.5 | 241.5 +/- 23.63 | 0.77 |
| BMI (kg/m^2^) ^a^ | 28.7 +/- 6.12 | 28.8 +/- 6.87 | 28.4 +/- 6.22 | 0.67 | 28.1 +/- 6.19 | 28.2 +/- 6.28 | 28.4 +/- 6.52 | 0.83 |
| Post menopause (n, %) ^b^ | 207, 77.8% | 271, 75.1% | 249, 64.7% | **<0.01** | 532, 72.0% | 492, 71.0% | 408, 67.6% | 0.19 |
| Stage at diagnosis (n, %) ^b^  Stage I  Stage II  Stage III/ IV | 139, 62.6%  71, 31.9%  12, 5.4% | 181, 60.3%  97, 32.3%  22, 7.3% | 160, 48.2%  146, 43.9%  26, 7.8% | **<0.01** |  |  |  | - |
| ER status (n, %) ^b^  Negative  Positive | 67, 26.9%  182, 73.1% | 99, 29.4%  238, 70.6% | 113, 30.9%  253, 69.1% | 0.57 | - | - | - | - |
| PR status (n, %) ^b^  Negative  Positive | 95, 38.5%  152, 61.5% | 120, 35.9%  214, 64.1% | 137, 38.1%  223, 61.9% | 0.78 | - | - | - | - |
| Hormone replacement therapy (n, %) ^b, f^  Yes  No | 109, 52.9%  97, 47.1% | 138, 52.1%  127, 47.9% | 129, 52.4%  117, 47.6% | 0.98 | 254, 49.6%  258, 50.4% | 231, 48.9%  241, 51.1% | 208, 52.5%  188, 47.5% | 0.54 |
| High blood pressure (n, %) ^b^  Yes  No | 100, 37.6%  166, 62.4% | 137, 38.4%  220, 61.6% | 123, 32.1%  260, 67.9% | 0.16 | 241, 32.7%  496, 67.3% | 234, 33.9%  456, 66.1% | 195, 32.4%  407, 67.6% | 0.82 |
| High blood cholesterol (n, %) ^b^  Yes  No | 106, 40.5%  156, 59.5% | 119, 33.9%  232, 66.1% | 124, 32.7%  255, 67.3% | 0.11 | 265, 36.6%  460, 63.5% | 259, 37.9%  425, 62.1% | 196, 33.1%  397, 66.9% | 0.19 |
| Comorbidities ^b^  Yes  No | 47, 18.1  213, 81.9% | 62, 18.0%  283, 82.0% | 66, 17.5%  311, 82.5% | 0.98 | 112, 17.1%  544, 82.9% | 120, 19.2%  504, 80.8% | 89, 16.0%  468, 84.0% | 0.32 |
| Ever alcohol consumption (n, %) ^b^  Non-drinkers in lifetime  Drinkers in lifetime | 42, 15.8%  224, 84.2% | 68, 19.1%  289, 80.9% | 64, 16.7%  320, 83.3% | 0.52 | 116, 15.8%  620, 84.2% | 104, 15.2%  582, 84.8% | 88, 14.7%  511, 85.3% | 0.86 |
| Marital status ^b^  Never married  Married  Widowed  Divorced/separated  Live with as if married | 23, 8.7%  154, 57.9%  41, 15.4%  44, 16.5%  4, 1.5% | 37, 10.4%  219, 61.7%  58, 16.3%  35, 9.9%  6, 1.7% | 24, 6.3%  235, 61.4%  58, 15.1%  57, 14.9%  9, 2.4% | 0.20 | 46, 6.2%  485, 65.8%  118, 16.0%  76, 10.3%  12, 1.6% | 35, 5.1%  439, 63.8%  126, 18.3%  77, 11.2%  11, 1.6% | 26, 4.3%  404, 67.3%  89, 14.8%  71, 11.8%  10, 1.7% | 0.63 |
| ^a^ Mean +/-standard deviation (SD) estimates were calculated for all continuous variables.  ^b^ Frequency and percentages were calculated for all categorical variables.  ^c^p-value compares breast cancer cases and healthy women by low, medium and high social support, comparison of means by ANOVA, and of frequencies by chi-square test and Fisher’s exact test.  ^d^ Low, medium and high social support was obtained by multiplying the number of friends with the number of times they were seen (where daily was assigned  a score of 30, once a week, 4, once a month 1 and less than once a month 0.5, and then it was analyzed in tertiles).  ^e^ Median and interquartile range  ^f^ only for postmenopausal women  -These are breast cancer specific variables and hence, no values available for healthy women | | | | | | | | |

**Supplementary Table 3.** Frequency of seeing close friends and relatives and mortality among breast cancer cases and population-based healthy women, multivariable adjusted HRs and 95% CIs

|  | **Breast cancer cases** | | | | **Population-based healthy women** | | | |
| --- | --- | --- | --- | --- | --- | --- | --- | --- |
| **Social support question (frequency of seeing close friends)** | **Nearly daily**  **(N=584)** | **About once a week**  **(n=341)** | **About once a month or less than once a month**  **(n=87)** | ***P_trend_*** | **Nearly daily**  **(N=955)** | **About once a week**  **(N=804)** | **About once a month or less than once a month**  **(N=277)** | ***P_trend_*** |
| **Total mortality** | 256 | 149 | 36 |  | 317 | 239 | 104 |  |
| Age-adjusted ^a^  Age and income-adjusted^b^  Multivariable-adjusted^c^ | 1.0 (Ref.)  1.0 (Ref.)  1.0 (Ref.) | 0.95 (0.78-1.16)  0.87 (0.70-1.09)  0.86 (0.68-1.10) | 0.87 (0.61-1.24)  0.82 (0.57-1.18)  0.71 (0.46-1.10) | 0.41  0.15  0.08 | 1.0 (Ref.)  1.0 (Ref.)  1.0 (Ref.) | 0.92 (0.78-1.09)  0.92 (0.77-1.10)  0.94 (0.79-1.13) | 1.14 (0.92-1.43)  1.10 (0.86 - 1.40)  1.09 (0.86-1.38) | 0.57  0.82  0.76 |
| **Breast cancer specific mortality** | 103 | 49 | 16 |  |  |  |  |  |
| Age-adjusted^a^  Age and income-adjusted^b^  Multivariable-adjusted^c^ | 1. (Ref.)   1.0 (Ref.)  1.0 (Ref.) | 0.84 (0.60-1.18)  0.73 (0.50-1.04)  0.70 (0.47-1.04) | 1.04 (0.61-1.75)  0.94 (0.54-1.63)  0.97 (0.54-1.76) | 0.65  0.28  0.31 |  |  |  |  |
| ^a^Age-adjusted: n=1012 for breast cancer cases, n=2036 for population-based healthy women  ^b^Age and income-adjusted: age (years), income [<10,000, 10,000-50,000, 50,001-100,000, >100,000 (reference)], n=858 for breast cancer cases, n=1869 for population-based healthy women  ^c^Multivariable-adjusted: age (years), smoking (life pack years), income [<10,000, 10,000-50,000, 50,001-100,000, >100,000 (reference)]. Only for breast cancer cases, cancer stage [stage I (the reference group), stage II, stage III/IV] was also included in the model, n=731 for breast cancer cases, n=1867 for population-based healthy women  -These are breast cancer specific variables and hence, no values available for healthy women | | | | | | | | |

**Supplementary Table 4.** Composite measure of social support and mortality among breast cancer cases and population-based healthy women, multivariable adjusted HRs and 95% CIs.

|  | **Breast cancer cases** | | | | **Population-based healthy women** | | | |
| --- | --- | --- | --- | --- | --- | --- | --- | --- |
| **Social support (close friends)** | **High social support * (N=385)** | **Medium social support ***  **(N=361)** | **Low social support * (N=266)** | ***P_trend_*** | **High social support ***  **(N=604)** | **Medium social support ***  **(N=693)** | **Low social support ***  **(N=739)** | ***P_trend_*** |
| **Total mortality** | 164 | 159 | 118 |  | 184 | 229 | 247 |  |
| Age-adjusted ^a^  Age and income-adjusted^b^  Multivariable-adjusted^c^ | 1. (Ref.) 2. (Ref.)   1.0 (Ref.) | 0.93 (0.75-1.16)  0.88 (0.69-1.16)  0.98 (0.75-1.27) | 0.91 (0.72-1.16)  0.84 (0.65-1.09)  0.90 (0.68-1.20) | 0.44  0.18  0.49 | 1.0 (Ref.)   1. (Ref.)   1.0 (Ref.) | 1.02 (0.84-1.24)  1.02 (0.83-1.25)  1.09 (0.88-1.34) | 1.11 (0.92-1.34)  1.07 (0.87-1.31)  1.12 (0.91-1.37) | 0.28  0.50  0.30 |
| Breast Cancer specific mortality | 70 | 57 | 41 |  |  |  |  |  |
| Age-adjusted ^a^  Age and income-adjusted^b^  Multivariable-adjusted^c^ | 1. (Ref.) 2. (Ref.)   1.0 (Ref.) | 0.91 (0.64-1.30)  0.79 (0.54-1.15)  0.90 (0.61-1.33) | 0.91 (0.62-1.34)  0.79 (0.52-1.19)  0.81 (0.52-1.26) | 0.60  0.22  0.34 | - | - |  |  |
| ^a^Age-adjusted: n=1012 for breast cancer cases, n=2036 for population-based healthy women  ^a^Age and income-adjusted: age (years), income [<10,000, 10,000-50,000, 50,001-100,000, >100,000 (reference)], n=858 for breast cancer cases, n=1869 for population-based healthy women  ^b^Multivariable-adjusted: age (years), smoking (life pack years), income [<10,000, 10,000-50,000, 50,001-100,000, >100,000 (reference)]. Only for breast cancer cases, cancer stage [stage I (the reference group), stage II, stage III/IV] was also included in the model, n=731 for breast cancer cases, n=1867 for population-based healthy women  ^*^ Low, medium, and high social support was obtained by multiplying the number of friends with the number of times they were seen  -These are breast cancer specific variables and hence, no values available for healthy women | | | | | | | | |
